# Supplementary material for: Are carbon emissions trading and green financial instruments synergistic? -Comprehensive quantitative research based on content analysis
Source: PLoS One. 2024 Mar 7;19(3):e0298601. doi: 10.1371/journal.pone.0298601 (PMC10919601; doi:10.1371/journal.pone.0298601)
Supplement: S1 Data — (ZIP) [file pone.0298601.s001.zip › Data/地级市Z_F工作报告.zip!/╔┬╬≈/▒a╝a╩╨/└┤╫╘╣1⁄2╓┌║┼í╛┬φ┐╦╩2╛▌═°í┐_2015.docx]

各位代表：

现在，我代表市人民政府向大会作工作报告，请予审议，并请市政协委员和其他列席人员提出意见。

2014年工作回顾

过去一年，面对复杂多变的宏观经济形势和繁重艰巨的改革发展任务，在市委的坚强领导下，市政府围绕做大总量、做优质量两大要务，主动适应新常态，全力以赴稳增长、促改革、调结构、惠民生，全市经济社会持续稳定健康发展，较好完成了市十四届人大四次会议确定的目标任务。全年实现地区生产总值1658.54亿元,增长10.8%；完成地方财政收入78.05亿元，同口径增长15.6%；城镇居民人均可支配收入增长10.7%，农民人均纯收入增长12.5%。

推进转型升级，经济质量明显提升。工业优势持续增强，全市规模以上工业实现增加值745.2亿元，增长12.2%，分别高于全国、全省3.9和0.9个百分点。净增规模以上工业企业66户，东岭集团总收入突破750亿元，在中国企业500强中前移15位，陕汽控股集团在宝产值突破百亿。工业园区建设加快推进，高新区跻身全国百强产业园区，眉县科技工业园跻身省级经开区，新增西凤酒业工业园等4个省级重点县域工业集中区。企业技术创新力度加大，新增省级企业技术中心3个。非公经济快速发展，占GDP比重达到50.07%。瑞科新材料在“新三板”成功上市，秦川机床集团实现整体上市，新材料高技术创业投资基金挂牌运营。农业现代化步伐加快，新栽苹果、猕猴桃、核桃等干鲜杂果25.7万亩，种植蔬菜90.3万亩，千阳县被命名为全国苹果现代化种植技术示范基地，陇县成为全省首个国家绿色核桃生产标准化示范区，麟游县跻身中国核桃之乡行列，太白县荣获中国绿色生态蔬菜十强县称号，扶风县被确定为全国放心农资下乡进村示范县。新建两千头奶牛场3个、500头标准化奶牛小区10个、万只以上肉鸡标准化养殖大棚27个。新增国家级现代农业示范区4个、省市级45个，全市家庭农场达到2173个。建设高标准基本农田34万亩，恢复改善灌溉面积10.4万亩，治理水土流失400平方公里。石头河引水工程建成通水，我市荣获第三批全国节水型社会建设示范区称号，全市13.6万农村人口解决了安全饮水问题。第三产业快速增长，法门寺景区成功晋级国家5A级旅游景区，太白山旅游区重新对外开放，新增4A级景区5个，全市共接待国内外游客4650万人次，实现旅游综合收入310亿元，分别增长28.3%和31.1%。新增限额以上商贸流通企业88户，成功举办了首届宝鸡青岛啤酒节和美食节，全市实现社会消费品零售总额535.59亿元，增长14%。“长安号”宝鸡货运专列顺利开通、常态运行，对丝绸之路沿线国家贸易增幅超过20%。我市被评选为中国城市信息化50强城市，渭滨区互联网产业园成功开园，岐山县荣获首批陕西省电子商务示范县称号。

突出项目建设，发展后劲持续增强。全年完成全社会固定资产投资2105.59亿元，增长26.1%。青岛啤酒宝鸡工业园一期、农夫山泉和龙源风电二期、高端装备石油产业园、千阳环保新材料、海螺型材、阜丰生物搬迁技改等一批重大产业项目建成，吉利汽车、西凤酒扩建技改、西部光谷光通信产业园等项目加快推进。法绛高速改扩建、国道342彬麟界至麟游县城段改扩建、省道212凤县县城过境段一期工程建成通车，连霍高速市区过境线、宝汉高速宝鸡段建设全面推进，宝鸡（凤翔）机场项目已上报国家申请立项。钛谷有色金属交易中心、西部煤炭交易物流园、红星美凯龙等商贸物流设施建成投用。全年落实招商引资项目846个，其中新引进10亿元以上项目19个，实现到位资金794.25亿元，增长22.5%。飞雳士安全轮胎、法士特中轻卡变速器、宝钛高尔夫运动器材等一批重大项目成功签约，招商引资项目数量、到位资金和重点项目投资额均创历年之最。

完善城镇设施，承载能力不断提高。重点区域开发取得突破性进展，南客站片区开发建设进展顺利，停车场、站前大道建成投用。重塑蔡家坡工作全面展开，高起点完成了蔡家坡地区总体规划修编，市政基础设施和公共服务设施项目建设全面推开。蟠龙新区基础设施建设快速推进，上塬路西线全线贯通，蟠龙大道路基和地下管网工程顺利完工。实施了161项城市建设管理提升工程，北环路、川陕路、石坝河街区改造等工程全部竣工，金陵三桥、陈仓区北新路建成投用，渭河代家湾段橡胶坝建成蓄水。市区南大门公园建成开放，射击射箭馆、游泳跳水馆建成投用，市体育场周边、高速公路出入口等城市节点区域整治效果明显。市区主要道路机械化清扫率达到40%。光明村、玉涧堡城中村改造拆迁任务全面完成，新东岭城市综合体建设稳步推进。5个省级重点示范镇建设开工项目82个，完成投资21.83亿元，12万农村人口实现进城落户。

大力改善民生，发展成果更加惠民。全市民生支出累计完成 197.4亿元，占财政支出的83.8%。在全省率先启动教育现代化先进县（区）创建工作，实施薄弱校改造等项目350个，市教育中心建成投用，太白县、千阳县建成国家义务教育发展基本均衡县。市中心医院内科楼、市口腔医院综合大楼等建成投用，新农合政策不断完善，大病医疗保险救助比例持续提高。单独两孩政策平稳实施，低生育水平保持稳定。县镇食品药品监管体制改革全面完成，被列为首批国家食品安全创建城市。陇县、凤翔县荣获中国民间文化艺术之乡称号，千阳县荣获全国文化先进县，石鼓山考古发掘被评为全国十大考古发现之一。我市运动员在第十七届亚运会上获得3枚金牌，实现零的突破，我市被命名为全国首批全民健身示范城市。新开工建设各类保障性住房42367套，31231套基本建成。完成87个老旧小区综合改造，解决了一批老百姓反映强烈的突出问题。城镇新增就业5.8万人，城乡居民养老保险参保人数达154.8万人。23万老年人享受高龄补贴，建成农村幸福院231个，陇县中心敬老院荣获全国农村五保供养工作先进单位，我市荣获全国和谐社区建设示范市。投入1.14亿元，连续第八年为全市残疾人办好了十件实事。1300户农村“三无户”移民新居主体完工，12.66万人实现脱贫。

加强生态建设，城乡环境更加优美。率先在全省推行大气污染防治网格化管理模式，控煤、降尘、治车成效显著，全年拆除燃煤锅炉953台，取缔储煤场地138个，削减燃煤210万吨，淘汰高污染车辆13383辆，全年市区空气质量二级以上天数达到242天，超过省考指标102天，空气质量优良达标率66%，位居关中各市第一。渭河水污染防治三年行动计划圆满收官，出境水质达到标准要求。凤县嘉陵江和太白县石头河建成国家湿地公园。农村环境综合整治走在全省前列，225个村的省级示范项目全面完成。全年完成造林绿化45.4万亩，千里绿色长廊建设经验在全省推广。市区新增绿地124.8万平方米，建成街头游园20处。国家生态市创建加快推进，群众对环境保护的满意度达到90.93%，比上年提高7.26个百分点。

深化改革创新，发展活力有效激发。行政审批制度改革扎实推进，累计下放、取消行政审批事项144项，减幅达26.3%。市县政府机构改革稳步推进，事业单位分类改革顺利开展。工商登记制度改革成效明显，全年新增市场主体28809户，增长35%。财税体制改革不断深化，市县两级部门预决算实现全面公开，全市“三公”经费支出下降15.96%。金融改革创新持续推进，在全省率先推出农村土地承包经营权抵押贷款等新产品，社会融资规模达到245.6亿元。千阳县、凤县农村土地承包经营权确权登记试点工作全面完成，全市集体林权证核发任务基本完成。医改工作再次被评为全省第一，公立医院改革得到国务院充分肯定，经营性文化事业单位转企改制取得突破。

与此同时，法治政府建设步伐加快，我市被命名为全省依法行政示范市。社会治安综合治理整体联动防控体系不断健全，流动人口、特殊人群和虚拟社会管理取得明显成效，人民群众对社会治安的满意率和政法队伍的满意度再获全省第一。全市安全生产形势持续稳定向好。党的群众路线教育实践活动扎实开展，网络问政、电视问政有力推进，为民服务水平进一步提高，群众对政府工作和干部作风的满意度分别比上年提高2.87和10.15个百分点。监察、审计、气象、档案、地震、地志、人民防空、民族宗教、国家安全、妇女儿童、侨务外事、红十字会等工作也取得了新的成绩。

各位代表，一年来，我们坚定信心、攻坚克难，全力冲刺全国文明城市，城乡居民文明素质全面提高，城市环境美丽宜人，被评为中国十佳最干净城市，入选2014最中国十大美食城市，成功入围第四届全国文明城市候选名单，16年来全市人民孜孜以求的创文梦想，即将成真，宝鸡的美誉度和影响力大幅提升。

一年来，我们周密部署、多方协调，高效有序承办了首次在中国大陆举行的第27届世佛联大会，受到中外嘉宾一致好评，得到世佛联、国家宗教局、中国佛教协会的充分肯定，让世界认识了宝鸡，让宝鸡走向了世界。

一年来，我们精心组织、密切协作，圆满成功举办了一届高水平、有特色、精彩难忘的陕西省第十五届运动会，受到省委、省政府奖励，并获得团体总分第一、金牌总数第二的历史最好成绩，全面展现了运动之城、和谐宝鸡的独特魅力。

一年来，我们凝心聚力、齐抓共管，举市一致打好创建国家生态园林城市攻坚战，创建工作顺利通过省级初审，自然生态和人居环境大幅改善，潺潺清水、茵茵绿地的生态品牌更加彰显，走出了一条经济发展和生态保护双赢的绿色发展之路。

各位代表，这些成绩的取得，凝聚着全市人民的心血汗水，凝结着包括驻宝部队在内方方面面的大力支持，展现出宝鸡崛起的勃勃生机。在此，我代表市政府，向各位人大代表、政协委员，向广大干部群众、离退休老同志，向各民主党派、工商联、无党派人士、各人民团体，向驻宝解放军和武警官兵、中省驻宝单位表示衷心的感谢和崇高的敬意！

当前，宝鸡正处在转型发展的关键时期，仍然面临着不少困难和问题：转型升级步伐较慢，经济增长动力单一；第三产业总量偏小，消费对经济增长的贡献还不够大；生产要素流动和聚集度不高，资源和产业优势没有充分发挥。对以上问题，我们将认真对待，不回避、不掩饰，努力加以解决。

2015年主要工作

今年是全面完成“十二五”规划的收官之年，也是全面深化改革的关键之年。当前，我国经济发展已经进入新常态，经济增长速度从过去的高速增长转向中高速增长，宏观经济环境已难以支撑过去那种依靠消耗资源和牺牲环境为代价来拉动经济高速增长的粗放型发展。但是，我国经济发展总体向好的基本面没有改变，国家陆续出台的一系列稳增长政策措施，继续实施积极的财政政策和稳健的货币政策，加快推进“一带一路”战略，强化对基础设施、战略性新兴产业和服务业的扶持力度，为我市加快发展带来机遇。我们必须正确认识新常态，准确把握新常态，主动适应新常态，抢抓新机遇，再添新举措，全力实现宝鸡经济社会新发展。

2015年政府工作的总体要求是：认真贯彻落实党的十八大和十八届三中、四中全会、中央经济工作会议及省、市委全会精神，坚定不移地做大总量、做优质量，坚持以增促稳、以快求进、转型提质、创新突破，深化改革开放，推进城乡一体，强化社会治理，提升民生保障，努力实现经济持续健康发展、社会大局和谐稳定。

主要预期目标是：地区生产总值增长12%左右，全社会固定资产投资增长20%以上，社会消费品零售总额增长13%，地方财政收入同口径增长12%，规模以上工业增加值增长14%左右，城镇登记失业率控制在4%以内，城乡居民人均可支配收入分别增长11%和12%左右，人口自然增长率控制在5.8‰以内，二氧化硫、化学需氧量、氨氮、氮氧化物排放量分别削减0.5%、1%、1.5%、6%，万元GDP能耗下降2.9%。

重点抓好九个方面工作：

一、加速推进转型升级，壮大工业经济实力

推进优势产业集群化发展。以建设国家新材料基地为目标，全面加快“宝鸡·中国钛谷”建设，重点抓好国家钛产业专利导航发展实验区、钛锆镍及其合金铸件产业化等项目建设，加快国家钛材产品质量监督检测中心建设，办好钛产业交易博览会。依托吉利汽车项目，规模化发展汽车及零部件产业，着力引进核心零部件配套企业，支持更多本地企业进入吉利汽车配套体系。延伸煤化工产业链，上半年宝氮整体搬迁技改一期项目建成投产，启动宝氮整体搬迁技改二期、长青能化甲醇二期项目。扎实推进西凤酒城、青岛啤酒宝鸡工业园续建项目，提升西凤酒、好猫烟市场占有率。继续支持大企业大集团加快发展，推进战略重组和行业整合，发展总部经济，培育壮大产业集群。

加快县域工业园区建设。以新型工业化为方向，发展县域工业园，完善配套功能，提升承载能力，加速产业聚集，年内建成标准化厂房5万平方米，鼓励小微企业和小项目入园发展。支持凤翔县创建省级高新技术产业开发区。年内县域工业园区产值突破1000亿元。

培育工业经济新增长点。加快发展风电、太阳能发电等战略性新兴产业，确保龙源风电后续项目、关山风电、英利光伏等如期并网发电。以渭滨区互联网产业园为重点，打造关天经济区互联网产业发展增长极。大力发展非公经济，创造更加公平的市场环境，做大非公经济总量，新发展一批科技型中小企业。加快两化深度融合步伐，大力改造提升传统工业，调整产品结构，全面提升企业竞争力。围绕智能机器人、绿色能源等前沿科技，依托秦川机床、宝钛集团等优势企业，催生新业态，培育新优势。

强化政策扶持服务。扎实开展“千名干部联系千户企业”活动，选派2000名干部深入2000户企业，采取帮办、陪办、代办等方式，重点帮助非公企业破难题、调结构，拓市场、促销售。全面落实中省市稳增长和区域采购奖励政策。抓住国家推进重点行业兼并重组的机遇，支持企业就地扩能改造，确保新增产能项目留在宝鸡。年内净增规模以上工业企业50户以上。

二、突出抓好项目建设，保持投资较快增长

加快重点项目建设。今年全市安排重点项目350个，计划完成年度投资600亿元以上，重点抓好产业项目，确保投资力度不减。全力抓好重大交通项目，加快宝兰高铁建设，确保连霍高速宝鸡过境线虢镇东至潘家湾段、蟠龙大道、西凤大道、国道344陇县县城过境段、陇县至关山旅游专线及宝麟铁路建成通车；开工建设长青工业园铁路专线、宝汉高速宝鸡至坪坎段、连霍高速过境线潘家湾至苟家岭段项目。做好关中城际铁路、宝鸡城市轻轨、货运南环线、合凤高速宝鸡段等重大交通项目前期工作，推动宝鸡（凤翔）机场项目取得突破性进展。

加大招商引资力度。坚持招大选优并重、内扩外引并举，强力推进招商引资工作。全年引进3—5个投资30亿元以上的项目，高新区、蔡家坡经开区、蟠龙新区各引进2个以上投资过20亿元的产业项目，各县区至少引进1个投资过10亿元的项目。做好产业选择，积极引进科技含量高、税收贡献大的项目。支持现有大企业大集团引进配套企业。面向东部地区，积极承接产业转移；面向西部地区，吸引新疆、青海、甘肃等地企业来宝设立总部或分公司，建设生活基地。全年招商引资到位资金达到700亿元。

创新投融资机制。探索建立多元化投入机制，采取发行债券、鼓励上市融资等多种模式,支持众力电器、晨光电器等科技型企业在“新三板”上市，促进成长性好的企业在区域股权交易市场挂牌融资。大力推广政府和社会资本合作模式，建立全市PPP项目库，积极引导民间投资进入基础设施建设、服务业等领域，力促民间投资占比持续提升，增速达到30%以上。

精心做好项目谋划。抢抓“一带一路”建设战略机遇，认真编制“十三五”规划及各类专项规划。围绕国家支持的重点领域，在铁路公路建设、重大水利工程、城市地下管网改造等方面，谋划一批管长远、利全局的重大基础设施项目。依托优势资源，在煤炭深加工、新材料、新能源等领域，谋划一批前瞻性强的重大产业项目，为经济发展聚潜能、增后劲。紧盯国家宏观政策，加大争取力度，年内争取中省资金150亿元以上。

三、加快重点区域开发建设，推进新型城镇化进程

做强高新区。全面推进国家创新型园区建设，提高创业创新能力。加快南客站片区开发建设进度，年内建成站南大道、市民广场。启动郭家崖等城中村改造，加快高新大道商圈建设，继续优化高新区发展环境。以科技新城为重点，着力实施水、电、气、暖等基础设施建设，增强承载能力和公共服务功能。加快法士特中轻卡变速器等项目建设，强化产业支撑，使高新区主要经济指标保持高速增长，城市面貌有大的提升，成为全市经济发展的重要引擎和商贸繁荣、现代气息浓郁的新城区。

重塑蔡家坡。高起点建设基础设施和配套服务设施，开工建设西宝中线东段和渭北西路，改造提升岐山大道、人民路商业街，启动水寨村、岐星村拆迁改造，美化提升蔡家坡高速出口和高铁广场周边区域。高标准新建蔡家坡高中、初中，妇幼医院基本建成，西机医院综合楼、蔡家坡国际影院、图书馆建成投用。推进飞雳士安全轮胎等项目建设，确保阜丰生物、海螺型材达产达效，西北机器铜合金、华强精密铸造项目建成投产。

建设新蟠龙。以打造生态城区为核心，加速蟠龙新区开发建设步伐，全面完成龙腾路、龙翔路等6条主干道路地下管网工程和道路续建工程，加快保障性住房建设，提高入住率，建设高端服务业和教育医疗公益设施，为新区发展聚集人气。强化产业招商，依托“光谷”项目，围绕光通信、电子类、高新技术类产业，做好相关配套企业落户，形成集群效应。

提升主城区。围绕完善功能、提升品位，大力实施城市建设管理提升工程，启动金台大道综合改造、东风路改造工程，加快新东岭城市综合体建设，完成渭河排污管网建设，加大城中村改造力度。加快虢镇与主城区融合步伐，支持陈仓区道北新城开发建设，完成虢蟠路北段跨铁路桥工程和虢蔡路一期拓宽改造，不断完善市政基础设施。深化城市管理体制改革，实行“条块结合、以块为主”的市、区分级管理模式，建立三级政府、四级管理的扁平化机制，提高城市精细化管理水平。

构建城镇群。支持凤翔“一区两城三基地”建设，加快撤县设区步伐；扎实推进陇县“一河两岸五区”建设，加快南岸新城开发和旧城改造。突出县域特色，高标准推进其它7个县城建设。抓好14个全国重点镇、5个省级重点示范镇、4个省级文化旅游街区和3个市级重点镇建设，打造示范样板，增强集聚能力，促进农业人口就近就地转移。

推进城乡一体化。围绕建设统筹城乡示范市，坚持项目优先支持、改革优先试点、资源优先配置，整合部门力量和项目资金，扶持渭河两岸新型工业园区、现代农业示范园区率先发展，生态景观示范区、新型农村社区同步推进，重点抓好眉县全县域和蔡家坡全镇域城乡一体化试点，把渭河沿线建设成为全市统筹城乡的核心区。

四、大力发展现代农业，推进农村经济持续发展

壮大特色产业规模。全面完成土地确权登记颁证各项任务，加快土地流转步伐，推动土地经营权向种养大户集中。深入开展高产创建，切实保障粮食安全，全年粮食总产保持在140万吨以上。以矮砧苹果和优质猕猴桃为重点，创建苹果矮砧试验示范点4个，新建果园10万亩。新栽核桃、花椒等干杂果10万亩。新发展设施蔬菜、秦岭高山蔬菜2万亩。加强监测监控，强化政策扶持，推动畜禽标准化、规模化养殖，促进奶业健康发展，建成飞鹤乳业4万只奶山羊基地。加快扶风阳晨10万头生猪、岐山大成公司10万套种鸡场建设，创建部省级示范场20个。

加快农业园区建设。发展壮大新型农业经营主体，年内眉县国家级猕猴桃批发交易中心建成投用，太白煜群建成国家级现代农业示范区，新增省级现代农业园区2个、市级20个，新认定省级家庭农场150个、市级200个。大力扶持10户农业产业化重点龙头企业加快发展，规范提升100个专业合作社经营水平，完善利益联结机制，加快农业产业化进程。

改善农村生产生活条件。开工建设麟游县长益庙水库，扎实推进冯家山水库、段家峡水库等大中型灌区续建配套节水改造项目。年内改造县乡公路100公里，新修改造基本农田4万亩，发展节水灌溉10万亩，解决10万农村人口安全饮水问题。坚持整体推进与精准扶贫相结合，全面推进“六大扶贫工程”，抓好89个整村推进项目，扶贫移民搬迁2000户，在全省率先解决农村“三无户”安全住房问题，年内实现11.56万农村贫困人口脱贫。

五、全力突破服务业，扩大第三产业规模

打造旅游发展新优势。加快王牌景区建设，以法门寺晋升为国家5A级旅游景区为契机，争取开拍电影《法门寺》，推出常态化演艺节目，丰富佛文化内涵，努力把法门寺打造成世界级旅游景点。强力推进太白山景区5A级创建，抓好关山草原、黄柏塬景区综合开发、金台观二期等15个重点旅游项目建设，打造一批精品旅游景区。加强与陕旅集团的合作，推动西周文化景区建设取得实质性进展。充分利用渭河治理成果，精心打造渭河“百里画廊”，建成“十里花海”“千亩荷塘”“万顷芦荡”等景观，让渭河百里生态、文化休闲景观长廊成为旅游发展新亮点。高标准规划建设岐山民俗村，提升乡村旅游品质。强化旅游宣传营销，大频率、高密度、全方位宣传推介，持续扩大旅游对外影响力。全年旅游总收入、接待国内外游客分别增长14.96%和13.95%。

加快发展夜间经济。全面实施发展夜间经济方案，加快建设高端购物中心、特色酒吧街、都市景观长廊等8个夜间经济示范点。以太阳市特色小吃城为重点，建设一批特色美食街区，推出一台体现宝鸡特色元素、具有现代气息的综艺节目，让游客品味宝鸡美食、畅游宝鸡美景、乐享宝鸡生活。配套实施政策扶持、区域亮化、公交延伸、商场延时、治安防控、舆论宣传等保障措施，整体联动，聚力推进，使夜间经济成为拉动消费的关键支撑。

大力发展新兴服务业。鼓励和支持大企业主辅分离，发展一批现代物流、仓储配送、广告营销、售后服务等企业，推动生产性服务业发展。加大对“关天之窗”、岐宝汇等电商企业，以及蟠龙智慧产业园的扶持力度，支持渭滨区创建全国电子商务发展示范基地，加快培育电子商务、网络营销等现代服务业，全年实现电子商务交易额160亿元。推进陈仓物流园区信息大厦、综合物流信息公共服务平台、宝商物流配送中心等项目建设。积极发展金融服务业，启动金融商务区建设，引导银行类金融机构加大对重点项目、中小微企业的信贷投入。培育发展娱乐健身、家政服务等生活性服务业，提高服务业的社会化、专业化和现代化水平。

促进城乡居民消费。启动实施第三轮“市场体系建设工程”，加快市区商贸流通基础设施建设，世纪荟萃、三迪国际、星钻国际等商业广场建成投用。突出农村商贸配送中心建设，继续抓好镇超工程、放心早餐、蔬菜进社区等惠民工程，完善流通体系，优化消费环境。办好“第四届名优商品博览会”等特色会展活动，全力拉动消费增长。大力促进商贸企业发展，新增限额以上企业30户。全年完成社会消费品零售总额603亿元。

六、强力推动美丽宝鸡建设，打造生态宜居环境

深入开展治污降霾。深化、细化、系统化开展治污降霾，依据环保新标准，倒逼火电、水泥、焦化等行业和燃煤锅炉使用单位提高煤炭品质，升级环保治理设施，完成各县区供热中心环保治理工程。进一步加大燃煤锅炉拆改力度，实现高污染燃料禁燃区燃煤锅炉、燃煤污染“双清零”目标。全面推行严格的扬尘治理措施，加强城市小道路硬化降尘工作，5000平方米以上的建设工地全部配备喷雾车，改变城市清扫保洁方式，对市区120条主次干道做到机械化清扫、精细化保洁。加速淘汰黄标车、老旧汽车，减少汽车尾气污染。积极完善网格化管理，加强区域联动联治，着力解决焚烧垃圾、秸秆等群众身边的大气污染问题。实行生态环境损害责任终身追究，强化社会监督，严厉处罚偷排偷放等违法行为，对履职不力导致严重污染的责任人依法追责。确保全年空气质量优良天数达到200天以上。

扎实推进绿化美化。继续实施绿色宝鸡“九大工程”和关中大地园林化建设，年内完成绿化45万亩、植树4000万株。加快连霍高速、宝平高速、西宝高铁沿线和城市出入口等重要节点的绿化提升，构建多层次、立体化的绿色景观。加大国家生态园林城市、国家生态市创建力度，支持眉县、岐山创建国家园林县城，太白、千阳创建国家生态县，凤县创建国家生态文明建设示范区，凤翔、扶风、麟游、陇县创建省级生态县，建成一批市级生态镇和生态村。

持续整治农村环境。以减少农村面源污染为重点，年内完成306个村连片整治示范项目。平原县重点巩固扩大集中连片整治成果，着力解决垃圾、生活污水就近处理问题。城区突出城郊结合部环境综合整治，着力打造街道靓点，有效消除城郊盲点。山区县将农村污染治理与造林绿化、造景美化相结合，全力打造生态靓丽、功能完善、干净整洁的美丽家园。

大力实施渭河流域治理。全面完成渭河支流口6座交通桥梁建设任务，确保6月底前两岸堤路全线贯通。启动新一轮渭河流域水污染防治三年行动计划,全面整治沿渭排污口，实现渭河宝鸡境内全面截污。实施小韦河、千河、金陵河等渭河支流综合治理，确保渭河水质稳定达标。抓好千渭之会等4个湿地公园建设，在全面规划的基础上，建设一批拦河坝、生态湿地水面景观。

七、切实强化民生保障，让群众共享更多发展成果

增强社会保障能力。率先开展“全民参保登记计划”，推进社保人群全覆盖。以加强市县镇村公共服务平台建设为抓手，切实解决好社会保障服务“最后一公里”问题。突出做好大学生及困难群体就业，做优“宝鸡技工”品牌。加大保障房及配套设施建设力度，落实棚户区改造补助资金，确保6月底前龙山雅居、虢镇茗苑、金陵湾二期等分配入住。适应老龄化进程，加快市县镇村四级老年文化活动中心、城市社区日间照料中心建设，年内全市20%的行政村农村幸福院建成投用。继续为残疾人办好十件实事，使残疾人共享改革发展成果。扎实推进军民共建活动，确保全国双拥模范城八连冠。

推动教育优先发展。深入开展中小学德育行动和优质教育普及行动，梯次推进教育创强工作。全面实施第二轮学前教育三年行动计划，完成21所幼儿园建设任务。实施270个薄弱学校改造、县城以上学校扩容等项目。着力发展职业教育，组建专业性职教集团，创新人才培养模式，服务地方经济发展。加快发展特殊教育，积极发展老年教育，构建终身教育体系。

提高医疗卫生服务水平。加快市全科医生培训基地、市精神卫生中心工程建设进度，提升改造县级医院，加大卫生人才培养力度。扎实开展卫生综合改革试点，支持凤翔县省级综合医改试点，深化公立医院改革，推进分级诊疗体系建设，探索建立现代医院管理制度，努力破解以药补医机制，进一步缓解看病难、看病贵问题。深化幸福家庭创建，促进全市人口实现均衡发展。启动健康城市建设，积极发展健康产业，提高全市人民健康水平。

发展文化体育事业。巩固文化体制改革成果，大力扶持发展文化产业。加强文物保护利用，抓好周原、石鼓山、北首岭等重点保护工程。开工建设市文化艺术中心等项目，承办好全国游泳冠军赛，积极参加全国首届青年运动会（原城市运动会），不断提升竞技体育水平，巩固全国全民健身示范城市创建成果。

八、全面深化改革开放，增强发展内生动力

深入推进各项改革。结合县区政府职能转变和机构改革，理顺条块管理体制，完善县区政府统一监管职责。实施机关事业单位养老保险制度改革，落实县以下公务员职务与职级并行、职级与待遇挂钩制度。切实做好公车改革，确保6月底前落实到位。积极推进镇村综合改革，提高基层组织社会治理和服务能力。加快国企改革步伐，做好困难企业改革脱困。深入推进商事制度改革，促进小微企业健康发展。继续深化财税体制、预算管理、专项资金管理、政府购买服务改革，促进财政管理科学化。按照责权利相统一的原则,完善园区管委会机制，激发园区发展活力。

提升科技创新能力。以国家创新型试点城市迎检验收为契机，实施好市级6个重大科技创新和创新型试点企业培育等工程，支持企业联合高校院所组建研发中心。积极引进高端人才，组织开展好两院院士宝鸡行、企业老总院校行、院校专家企业行等活动，推进产学研合作，加速科技成果转化。

拓宽对外开放空间。围绕打造丝绸之路战略新枢纽，支持有条件的企业在境外投资办厂，年内挂牌成立砖机、纺织、猕猴桃、苹果等一批市级出口基地。鼓励汽车制造、石油装备、轨道交通等企业建立海外营销网络和售后服务体系，引进专业外贸公司5-8户。积极推进宝鸡东站迁建，加快阳平铁路货运站建设，推动物流产业加速发展。充分利用“长安号”列车，扩大对外出口，加快向西开放步伐，提高经济外向度。

九、着力加强社会治理，保持社会和谐稳定

夯实基层基础。全面落实城市社区布点规划，深入实施社区建设提升工程，持续抓好和谐示范社区创建工作，积极开展矛盾纠纷排查化解，不断健全“一厅、一网、一热线”服务模式，实现市区、县区、社区网络互联，提升社区信息化管理水平。突出重点人群，发展专业化便民服务，夯实社会管理基层基础。加强政策指导和督促检查，确保全面完成第九次村民委员会换届选举工作。加强物业管理，完善物业保修金制度，积极推行招投标制度，推进物业管理市场化进程。

推进平安建设。深入推进平安宝鸡建设，健全立体化治安防控体系，严格落实社会稳定风险评估机制，依法打击非法集资等违法犯罪活动，全面加强禁毒和公共安全管理，打造最安全城市。深入实施“六五”普法规划，加快依法治市进程。加强流动人口和特殊人群的服务管理，对失业、失亲、失独等人群给予更多的关爱。扎实做好源头预防、积案化解工作。强化属地管理责任，加强市场和农产品质量安全监管；积极开展全国食品安全城市创建活动，确保舌尖上的安全。落实安全生产监管责任和企业主体责任，有效预防各类安全事故发生。

加强政府自身建设

打铁还需自身硬。新形势、新任务对政府工作和自身建设提出了新的要求。我们将进一步加强政府治理体系和行政能力建设，全面提高政府公信力、执行力和创造力，以务实高效的作风服务于民，以勤政清廉的形象取信于民，努力打造人民满意政府。

坚持法治理念，推进依法行政。坚持法无授权不可为，法定职责必须为，打造法治政府。深化政务公开，全面落实政务信息发布制度，提高工作透明度，保障群众知情权。自觉接受人大工作监督和法律监督以及政协民主监督，认真办理人大代表建议和政协委员提案，更加重视公众监督、舆论监督，继续办好市政府门户网站“在线访谈”栏目和公民代表走进市政府活动，将政府的一切行为置于法律框架内和人民监督下，让权力在阳光下运行。

坚持改革创新，加快职能转变。深化行政审批制度改革，继续加大简政放权力度，市级部门行政审批事项再减少三分之一以上，向县区政府下放行政权力的同时，认真做好中省下放行政审批事项的承接实施。按照权责一致原则，推进各级政府及工作部门公布权力清单，制定负面清单，细化运行流程。加强政府绩效管理，大力推进并联审批、网上审批，加大行政问责、行政过错责任追究力度，建立健全工作绩效公开制度，不断提高行政效能。

坚持执政为民，促进作风改进。持之以恒纠正“四风”，推动群众路线教育实践活动成果常态化、长效化。在政府系统大力倡导低调务实不张扬、埋头苦干抓落实的作风，对确定的目标、制定的政策、部署的任务，以“钉钉子”精神，一抓到底，务求实效。按照“三严三实”的要求，从严管理干部，深化拓展百姓问政、万名群众评议百名科长等活动，坚决纠正为政不为、懒政、乱作为、慢作为行为，不断提升为民服务水平。大兴艰苦奋斗之风，严格执行八项规定和党政机关厉行节约反对浪费条例，坚持过紧日子，厉行节约，反对浪费，规范各类检查、评比、达标、表彰活动，腾出更多资金改善民生。

坚持清正廉洁，提升政府形象。严格执行党风廉政建设责任制和廉洁从政有关规定，坚持用制度管权管事管人，加大对工程建设、招标采购、项目管理、土地出让等重点领域的廉政风险防控力度。加强廉政教育，各级公务人员都要讲规矩、知敬畏、守底线。加大对群众身边不正之风和腐败问题查处力度，切实解决以权谋私、与民争利和损害群众利益等问题，树立为民、务实、清廉的政府形象。

各位代表，新起点孕育新希望，新常态要有新作为。面对百舸争流、千帆竞发的新征程，让我们在市委的正确领导下，解放思想、改革创新，锐意进取、真抓实干，为打造关天副中心、建设和谐新宝鸡，走在建设“三个陕西”前列做出新的更大的贡献！
